# Protective Effect of GIP against Monosodium Glutamate-Induced Ferroptosis in Mouse Hippocampal HT-22 Cells through the MAPK Signaling Pathway

**DOI:** 10.3390/antiox11020189

**Published:** 2022-01-19

**Authors:** Jiwon Ko, Soyoung Jang, Wookbong Kwon, Si-Yong Kim, Soyeon Jang, Eungyung Kim, Young-Rae Ji, Sijun Park, Myoung-Ok Kim, Seong-Kyoon Choi, Dong-Hyung Cho, Hyun-Shik Lee, Su-Geun Lim, Zae-Young Ryoo

**Affiliations:** 1 BK21 FOUR KNU Creative BioResearch Group, School of Life Sciences, Kyungpook National University, Daegu 41566, Korea; esther7949@naver.com (J.K.); wkdthdud21@naver.com (S.J.); pau26@naver.com (S.-Y.K.); jangsssso@naver.com (S.J.); youngrae.ji@nih.gov (Y.-R.J.); dhcho@knu.ac.kr (D.-H.C.); leeh@knu.ac.kr (H.-S.L.); 2 Core Protein Resources Center, DGIST, Daegu 42988, Korea; 6funny@naver.com (W.K.); cskbest@dgist.ac.kr (S.-K.C.); 3 Division of Biotechnology, DGIST, Daegu 42988, Korea; 4 Department of Animal Science and Biotechnology, Kyungpook National University, Sangju-si 37224, Korea; wjddn5460@naver.com (E.K.); ok4325@knu.ac.kr (M.-O.K.); 5 Section on Sensory Cell Regeneration and Development, Laboratory of Molecular Biology, National Institutes of Health, Bethesda, MD 20892, USA; 6 School of Life Science, Kyungpook National University, Daegu 42988, Korea; mooook15@naver.com; 7 Brain Science and Engineering Institute, Kyungpook National University, Daegu 42988, Korea

**Keywords:** monosodium glutamate, neurotoxicity, ferroptosis, oxidative stress, cellular oxidative homeostasis, mouse hippocampal HT-22 cells, glucose-dependent insulinotropic polypeptide, mitogen-activated protein kinase

## Abstract

The effect of glucose-dependent insulinotropic polypeptide (GIP) on cells under oxidative stress induced by glutamate, a neurotransmitter, and the underlying molecular mechanisms were assessed in the present study. We found that in the pre-treatment of HT-22 cells with glutamate in a dose-dependent manner, intracellular ROS were excessively generated, and additional cell damage occurred in the form of lipid peroxidation. The neurotoxicity caused by excessive glutamate was found to be ferroptosis and not apoptosis. Other factors (GPx-4, Nrf2, Nox1 and Hspb1) involved in ferroptosis were also identified. In other words, it was confirmed that GIP increased the activity of sub-signalling molecules in the process of suppressing ferroptosis as an antioxidant and maintained a stable cell cycle even under glutamate-induced neurotoxicity. At the same time, in HT-22 cells exposed to ferroptosis as a result of excessive glutamate accumulation, GIP sustained cell viability by activating the mitogen-activated protein kinase (MAPK) signalling pathway. These results suggest that the overexpression of the GIP gene increases cell viability by regulating mechanisms related to cytotoxicity and reactive oxygen species production in hippocampal neuronal cell lines.

## 1. Introduction

Monosodium glutamate (MSG), the sodium salt of the non-essential amino acid, glutamic acid, is a well-known food flavour enhancer that is naturally present in many foods, including tomato, cheese, and meat [1]. Although MSG consumption has increased globally, excessive MSG intake has been associated with an increased risk of various diseases, such as obesity, diabetes and asthma [2]. The negative effects of MSG were revealed to the public due to Chinese restaurant syndrome, characterised by headache, throbbing of the head, dizziness, facial pressure, burning or tingling sensations over parts of the body and back pain [3]. Glutamate is the main excitatory neurotransmitter in the mammalian central nervous system. Glutamate toxicity plays a critical role in causing neuronal dysfunction and oxidative stress in many acute and chronic neurological diseases by regulating cell survival and death [4]. The accurate regulation of cell survival and death is crucial for homeostasis, cell–cell interaction and development [5].

Initially, most programmed cell deaths were broadly divided into autophagy, apoptosis and necrosis [6]. More recently, however, many novel types of cell death have been identified; these include parthanatos, necroptosis and ferroptosis. These regulated cell deaths have different morphological, physiological and functional hallmarks [7]. The activation of cortical neurons in the brain of patients with progressive multiple sclerosis (MS) indicates that neurodegeneration occurs via necroptosis rather than apoptosis [8]. Parthanatos, i.e., poly (ADP-ribose) polymerase 1 (PARP1)-dependent cell death, is a cell death signalling pathway in which excessive oxidative damage to DNA leads to the overactivation of PARP. PARP inhibitors for decreasing parthanatos play a neuroprotective role in ischaemic stroke [9,10]. Ferroptosis, a new type of programmed cell death that is dependent on iron and lipid peroxidation-induced necrosis, has received noteworthy attention due to its impact on human health and disease [11]. Ferroptosis is a non-apoptotic form of cell death that causes lipid damage and membrane permeabilisation. When lipid reactive oxygen species (ROS) and lipid oxidation targets, such as polyunsaturated fatty acids (PUFAs), accumulate in the cell membrane, the cells ultimately undergo morphological changes [11,12]. It is well known that the plasma membrane of apoptosis-induced cells is blebbing, forming apoptotic cell bodies and fragmenting their nuclei [13]. Notable morphological phenotypes observed during ferroptosis include outer mitochondrial membrane rupture, reduced mitochondrial volume and disorganised cristae [11]. This ferroptosis type of cytotoxicity could be triggered by numerous factors, one of which is glutamate in neurons [14].

Glutamate plays a major role in several neurodegenerative diseases, such as Parkinson’s disease, Huntington’s disease, Alzheimer’s disease, and seizures [15,16]. However, the degree of glutamate-induced toxicity differs depending on the cell type and the presence of glutamate receptors [17]. On the other hand, only a few studies have explained the relationship between some specific genes and glutamate-induced neuronal diseases. Glutamate is the critical excitatory neurotransmitter in the central nervous system [18]. It stimulates postsynaptic neurons by inducing an influx of calcium and sodium ions. However, excessive extracellular glutamate inhibits cystine import via the cystine/glutamate antiporter system Xc^−^ [19]. This triggers a series of events, including antioxidant glutathione (GSH) depletion, lipoxygenase activation, ROS accumulation and calcium (Ca^2+^) influx, eventually causing oxidative stress [20]. Due to the damage resulting from excessive ROS accumulation, the type of glutamate-induced neuronal cell death was initially regarded as apoptosis or necrosis [21]. In various features of morphological and biochemical characteristics, ferroptosis is classified as a new regulated cell death separate from apoptosis. For example, apoptosis is characterised by membrane shrinkage, chromatin condensation, DNA fragmentation, and caspase activation [22], but ferroptosis adopts a rounded shape before cell death and no cytoplasmic and organelle swelling, or plasma membrane rupture [23].

Although ferroptosis has been actively studied in recent years, the precise details of how and which transcriptional or translational effectors cause ferroptosis remain largely unknown. The initiation and progress of ferroptosis, mainly induced by amino acid metabolism, iron metabolism, lipid peroxidation and lipid ROS production, are modulated by multiple signalling pathways and factors [24]. Superfluous ROS accumulation can lead to cell damage and induce cell death through the modulation of the following intracellular signalling pathways that include mitogen-activated protein kinases (MAPKs): the extracellular signal-regulated kinase (ERK), c-Jun N-terminal kinase (JNK) and p38 MAPK pathways [25]. Redundant glutamate, which produces the ferroptosis pathway, also causes the phosphorylation of MAPKs, including ERKs, JNKs and p38 [26].

Glucose-dependent insulinotropic polypeptide (GIP) was first identified as an incretin hormone secreted by intestinal endocrinal cells. Although GIP is primarily produced in the intestine, it can play a neuroprotective role in the brain due to blood–brain barrier (BBB) permeability [27]. In a Parkinson’s disease model, motor activity was found to be normalised, dopaminergic neurons were protected, synapse numbers and dopamine levels were maintained and chronic inflammation and mitochondrial damage were reduced upon treatment with GIP [28]. In addition, GIP enhanced neurogenesis by promoting progenitor cell proliferation in the adult mammalian brain and improved motor function in GIP transgenic (Tg) mice [29,30]. Moreover, GIP analogues were found to improve synaptic plasticity, the long-term potentiation (LTP) of neuronal synaptic transmission and memory formation [31]. The neuroprotective effects of GIP were mainly studied using pharmacological agents, such as GIP analogues or agonists. Some studies have already assessed whether GIP could be used as a novel therapeutic option for delaying apoptosis. However, their results only emphasise the function of GIP in terms of several critical homeostatic abilities without any stimulation [32]. Thus, the present study is the first to assess the self-recovering ability of native GIP in mouse hippocampus cells under fatal cellular oxidative stress induced by glutamate, a specific cell death inducer.

## 2. Materials and Methods

### 2.1. Cell Culture and Reagents

The mouse hippocampal cell line HT-22 was cultured in Dulbecco’s modified Eagle’s medium (DMEM) (Gibco, Auckland, New Zealand) supplemented with 10% foetal bovine serum (FBS) (Gibco) and 1% streptomycin–penicillin (Gibco) at 37 °C in the presence of 5% CO_2_. HT-22 cells were obtained from (Sigma-Aldrich, St. Louis, MO, USA) and sub-cultured once every 2 days. For stimulation, the cells were seeded at a concentration of 1.0 × 10^5^ cells/mL and incubated overnight in a 60 mm dish (SPL Life Science, Pocheon-si, Korea) in 4 mL of DMEM. Then, the cells were treated with 5 or 10 mM glutamate (Sigma).

### 2.2. Establishment of GIP-Overexpressing Cell Lines

Mouse GIP was cloned into the pEGFP-N3-NSE vector digested with HindIII and NotI. The correct sequence was verified and amplified. HT-22 cells (1 × 10^5^ cells/well) were plated on six-well plates and transfected with a recombinant plasmid using Lipofectamine 2000 (Invitrogen, Carlsbad, CA, USA). Following transfection, the cells were selected by assessing resistance to 800 μg/mL neomycin (G418) (Invitrogen, Carlsbad, CA, USA) for 2 weeks.

### 2.3. Cell Counting Kit-8 (CCK-8) Assay

CCK-8 (Dojindo, Kumamoto, Japan) was used to assess cell viability and proliferation. HT-22 cells were seeded in 96-well plates at a density of 5000 cells per well for 24 h. Then, 0–5, 10, 15 and 20 mM glutamate was added to the culture medium seeded with HT-22 cells and incubated for 24 h. Cell proliferation was assessed every 24 h. Relative data are shown as a percentage of the control, which was not treated with glutamate. Cell viability and proliferation were evaluated by measuring absorbance (450 nm) using a microplate reader (BGM RABTECH, Ortenberg, Germany) after adding the CCK-8 reagent (10 μL/well).

### 2.4. RNA Isolation and Quantitative Reverse Transcription Polymerase Chain Reaction (qRT-PCR) Analysis

Cells were collected, and total cellular RNA was extracted using TRI Solution (BSK-BIO, Daegu, Korea), according to the manufacturer’s instructions. cDNA was synthesised with 1 μg of total RNA using the 1st Strand cDNA Synthesis Kit (TaKaRa Bio Inc., Shiga, Japan). Real-time qPCR was performed using StepOnePlus (Applied Biosystems, Foster City, CA, USA) with SYBR Premix Ex Taq (Takara Bio Inc., Otsu, Shiga, Japan). The threshold cycle (Ct) values obtained for each reaction were normalised using the GAPDH Ct values. The sequences of primers used for real-time qRT-PCR are outlined in Table 1. All reactions were performed in triplicate.

### 2.5. Western Blotting

Cells were lysed with lysis buffer containing 20 mM Tris–HCl (pH 7.4), 420 mM NaCl, 2 mM EDTA, 10 mM MgCl2, 1% Triton X-100 (Thermo Fisher Scientific, Waltham, MA, USA), 10% glycerol and 1% phosphatase and protease inhibitor cocktail (Complete EDTA-free; Roche, Basel, Switzerland). The debris was then removed from total cell lysates by centrifugation (12,000 rpm for 15 min at 4 °C), separated using SDS–PAGE (8–15%) and electroblotted onto PVDF membranes (Millipore Sigma, Billerica, MA, USA). Following this, the membranes were blocked using 5% skimmed milk (MB Cell, Los Angeles, CA, USA) or 5% bovine serum albumin (Amresco, Solon, OH, USA) in Tris-buffered saline with Tween (TBS-T; containing 25 mM Tris–HCl (pH 7.4), 150 mM NaCl and 0.1% Tween 20). Next, the membranes were incubated at 4 °C overnight with primary antibodies. After washing thrice (5 min each) with TBS-T, the membranes were incubated with secondary antibodies for 2 h at room temperature (RT). After washing thrice (5 min each) with TBS-T, the protein level was determined by detecting chemiluminescence in response to Clarity Western ECL Substrate (Bio-Rad Laboratories, Hercules, CA, USA). Finally, specific bands on the membranes were detected using the Davinch-Chemi Chemiluminescence Imaging System (CoreBio, Seoul, Korea). Western blotting was performed using antibodies against anti-GIP (GTX55639; GeneTex, Irvine, CA, USA), Bcl-xL (#2764), Bcl-2 (#3498), Bax (#2772), Bak (#12105), survivin (#2808), cleaved PARP (#5625), PARP (#9532), cleaved caspase-9 (#9509), caspase-9 (#9508), cleaved caspase-3 (#9661), caspase-3 (#9662), phospho-Rb (ser807/811) (#8516), Rb (#9313), E2F-1 (#3742), cyclin D1 (#2922), cyclin E1 (#20808), cyclin B1 (#4135), CDK4 (#12790), CDK6 (#3136), CDK2 (#2546), FTH1 (#4393), p44/42 MAPK (Erk1/2) (#4695), phospho-JNK (Thr183/Tyr185) (#4668), JNK (#9252), phospho-p38 MAPK (Thr180/Tyr182) (#4511), p38 MAPK (#8690), p-B-Raf (#14814), B-Raf (#2696), p-MEK (#9154), MEK (#9126), p-MKK3/6 (#12280), MKK3 (#8535), #MKK6 (#8550), GAPDH (#5174; Cell Signaling Technology, Danvers, MA, USA), cyclin A1 (ab53699), Nox1 (ab131088; Abcam), SLC7A11 (MA5-35360, Thermo Fisher Scientific), nuclear factor erythroid 2-related factor (Nrf2) (sc-365949) p-ERK (sc-7383) and Gpx-4 (sc-166570; Santa Cruz Biotechnology, Dallas, TX, USA).

### 2.6. Flow Cytometric Analysis

For cell cycle analysis, HT-22 cells (in a 60 mm cell culture dish) were treated with glutamate for 24 h. After the cells were harvested, they were washed twice with Dulbecco’s phosphate-buffered saline (DPBS) (Gibco) and fixed using 100% ethanol (Merck, Kenilworth, NJ, USA) at 4 °C for 1 h. Following this, the cells were washed twice with DPBS, resuspended in DPBS containing 100 μg/mL RNase A (Thermo Fisher, Waltham, MA, USA) and incubated at 37 °C for 1 h. Then, the cells were stained using 1000 μg/mL propidium iodide (PI) (Invitrogen, Carlsbad, CA, USA) for 30 min. To assess the degree of cell death, the Annexin V-FITC/PI Apoptosis Detection Kit (Thermo Fisher) and BD Accuri Flow Cytometer (BD Biosciences, San Jose, CA, USA) were used, in accordance with the manufacturer’s instructions.

To measure the intracellular ROS levels, HT-22 cells were treated with glutamate (0 or 5 mM) for 6 h. Subsequently, the cells were stained for 10 min at 37 °C with 5 μM CM-H2DCFDA (DCFDA, Molecular Probes, Eugene, OR, USA) for detecting cellular ROS and with 5 μM MitoSOX (Thermo Fisher) for detecting mitochondrial ROS. The cells were harvested and washed with PBS. Following this, the cells were resuspended in 500 μL PBS for flow cytometric analysis using the FACS Verse System (Becton-Dickinson, Mountain View, CA, USA).

To assess lipid peroxidation fluorescence, HT-22 cells were treated with glutamate (0 or 5 mM) for 6 h and then supplemented with 2.5 µM C11-BODIPY^581/591^ (Thermo Fisher), incubated for 30 min and analysed using the FACS Verse System (Becton-Dickinson, Mountain View, CA, USA).

### 2.7. Measurement of MDA

An amount of 2 × 10^7^ HT-22 cells were treated with 5 mM glutamate harvested after 6 h into 1 mL PBS. Samples were sonicated and the homogenate was stored on ice. Malondialdehyde (MDA) was evaluated by a TBARS Assay Kit (Cayman Chemical, Ann Arbor, MI, USA) according to the manufacturer’s instructions.

### 2.8. Immunocytochemistry

Attached cells on chamber slides were fixed with 4% paraformaldehyde for 15 min at RT and permeabilised with 0.5% Triton-X in PBS for 15 min. The cells on cover slips were incubated with anti-GIP mAb (4 μg/mL) at 4 °C overnight and then incubated with 10 μg/mL of FITC-conjugated goat anti-mouse secondary antibodies at RT for 1 h. Cover slips were mounted with DAPI-containing mounting solution for observation. All images were acquired using LAS AF software (Leica Microsystems Inc., Wetzlar, Germany, https://www.leica-microsystems.com, accessed on 20 November 2020).

### 2.9. Statistical Analysis

Data are presented as the means ± standard deviations (SDs). All the experiments were performed a minimum of three times. Statistical significance was determined using the unpaired-sample two-tailed Student’s t test. Excel software was used to calculate *p*-values (* *p* < 0.05, ** *p* < 0.01 and *** *p* < 0.001 were considered statistically significant).

## 3. Results

### 3.1. Glutamate Treatment Reduces the Viability of HT-22 Cells in a Dose-Dependent Manner

**Figure 1:**
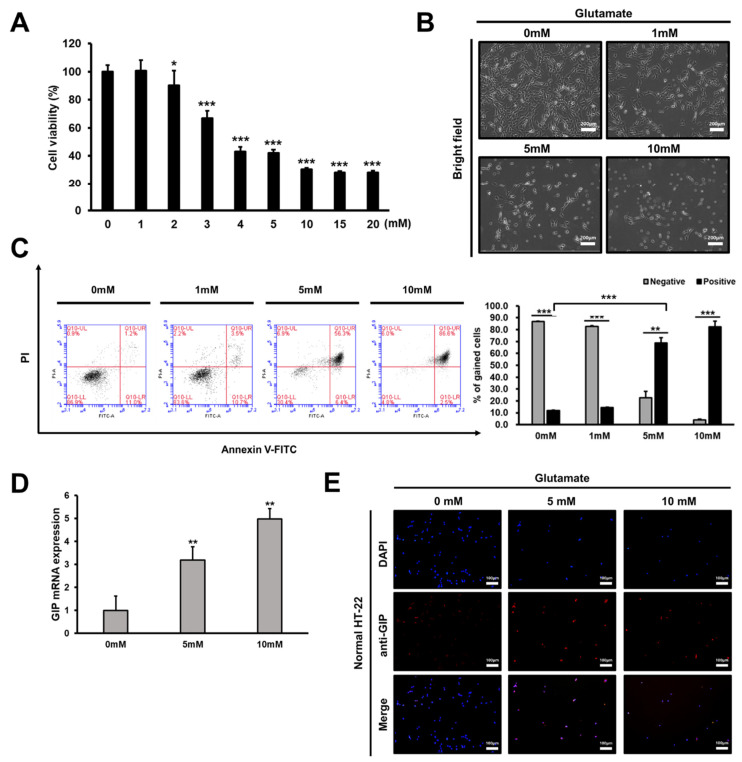
Effects of glutamate-induced neuronal cell death in mouse hippocampal HT-22 cells. (**A**) The viability of HT-22 cells treated with the indicated concentrations of glutamate for 24 h was measured by the CCK-8 assay. (**B**) Images of HT-22 cell morphologies were obtained using a microscope. Magnified images of cells treated with 0, 1, 5, and 10 mM glutamate for 24 h. (**C**) The damage caused by glutamate-induced neuronal toxicity under the indicated glutamate treatment conditions. The number of cells stained with annexin V–PI after glutamate treatment was assessed by flow cytometry according to our gating line (left) and the normalised percentage of gained cells that died by excessive glutamate (right) in HT-22 cells. (**D**,**E**) HT-22 cells were treated with the indicated concentrations of glutamate to verify the basal level of GIP. Following this, the mRNA (**D**) and protein (**E**) levels of GIP were evaluated using qPCR and immunofluorescence assays, respectively. Red fluorescent protein fluorescence was observed to determine the basal level of GIP in normal HT-22 cells. * *p* < 0.05, ** *p* < 0.01 and *** *p* < 0.001 compared with the corresponding untreated control.

To confirm the effect of GIP on glutamate-induced neuronal cytotoxicity [33], the optimal concentration of glutamate required to induce cell death in mouse hippocampal HT-22 cells was determined. Initially, the effects of glutamate treatment at various dosages on HT-22 cell viability were assessed using the CCK-8 assay. Compared with untreated cells, there was no significant difference in the viability of cells treated with up to 2 mM glutamate. However, cell viability decreased by approximately 50% upon treatment with 5 mM glutamate; this percentage decreased to less than 30% upon treatment with 10 mM glutamate (Figure 1A). Thus, we selected 5 or 10 mM glutamate to trigger HT-22 cell death for further experiments. After treatment with these dosages of glutamate, remarkable changes were noted in the number of live cells and cell morphology. Upon increasing the concentration of glutamate, the HT-22 cell number decreased (Figure 1A), and the cells turned to a round shape without their unique branches (Figure 1B). Next, flow cytometric analysis was performed with annexin V–PI double staining to confirm cell death in more detail. Cells that were positive for annexin V and/or PI were considered dead cells. Conversely, cells that were negative for both annexin V and PI were considered as live cells. Upon double staining with 5 mM glutamate, the death of HT-22 cells dramatically increased by up to 60% (Figure 1C) after 24 h of glutamate treatment.

In relation to the toxicity of glutamate, which causes cell death, we tried to find a specific factor that could alleviate it. Meanwhile, GIP analogues have been reported to protect against the amyloid-induced impairment of synaptic plasticity in the hippocampus [34]. Additionally, chronic stress can induce neuronal cell death in the mouse hippocampus [35]. Hence, we first verified GIP expression patterns in normal HT-22 cells by treatment with specific concentrations of glutamate. GIP mRNA expression levels gradually increased upon treatment with a high concentration (5 or 10 mM) of glutamate (Figure 1D). The protein levels also increased in immunofluorescence assays with DAPI and GIP-specific antibodies. Although GIP protein levels were not remarkable in normal HT-22 cells, they increased in response to glutamate in a dose-dependent manner (Figure 1E). GIP mRNA and protein levels increased under glutamate-induced neuronal damage conditions in normal HT-22 cells. These results suggest that GIP expression positively correlates with the severity of glutamate-induced toxicity response and that GIP is related to glutamate-induced neuronal damage.

### 3.2. Association between GIP Overexpression and Glutamate-Induced Neuronal Cell Death

**Figure 2:**
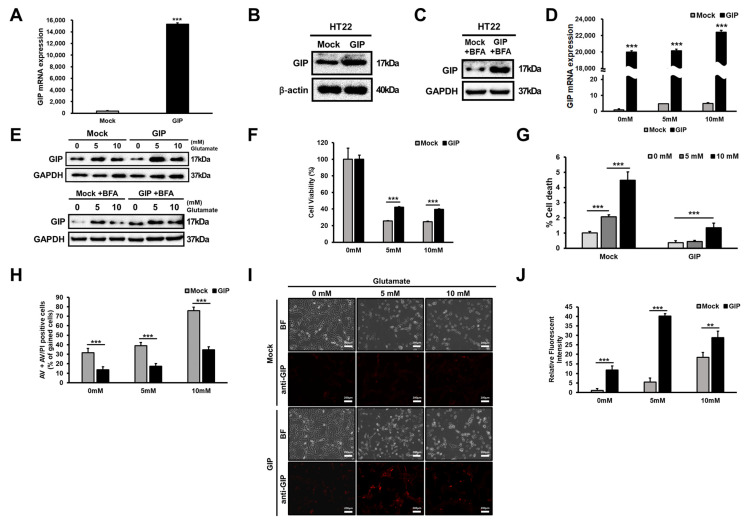
Increase in GIP expression upon glutamate treatment enhances neuronal HT-22 cell viability. (**A**) GIP mRNA expression levels were determined by qRT-PCR after establishing GIP-overexpressing HT-22 cells. Western blot analysis of GIP protein levels under normal conditions (**B**) and after treatment with BFA for 3 h (**C**). (**D**) GIP mRNA expression levels were checked upon treatment with the indicated concentrations of glutamate by qRT-PCR. The graph shows the expression level of GIP relative to that of GAPDH. (**E**) Western blot analysis of GIP protein levels. The cells were treated with different concentrations of glutamate (6 h) after treatment with BFA (3 h) or not. (**F**) The CCK-8 assay was performed to compare the cell viability between mock and GIP-overexpressing HT-22 cells after glutamate treatment (24 h) (same as previous data). (**G**) Effect of glutamate-induced cell death was determined by PI staining. (**H**) The degree of glutamate-induced cell death was evaluated by counting the number of double positive-stained cells by flow cytometric analysis. (**I**) HT-22 cells were incubated with glutamate in each group for 24 h. GIP expression was observed using antibodies for immunofluorescence assays, and bright field images were captured using a phase-contrast microscope (magnification, 20×). (**J**) The relative fluorescence intensity of GIP in the cell images was measured using ImageJ. Data are presented as the means ± SDs (*n* = 3). ** *p* < 0.01 and *** *p* < 0.001.

As shown in Figure 1D,E, the expression level of GIP increased in glutamate-treated normal HT-22 cells. Thus, we hypothesised that increased GIP is involved in the regulation of glutamate-induced neuronal cell death. Although a few GIP analogues are used for various clinical cures, most modified GIP analogues show different GIPR-binding affinities, unlike native GIP [36]. Therefore, we established a stable cell line overexpressing GIP with the neuron-specific enolase promoter in hippocampal HT-22 cells to assess the role of GIP under glutamate-induced neuronal damage conditions. We assessed the expression levels of GIP by measuring the mRNA levels in mock and GIP-overexpressing cells (Figure 2A). The protein level was also a difference between the mock and GIP-overexpressing group but it was smaller than we expected (Figure 2B). To prevent a problem in the process of quantification with a housekeeping gene, we attempted quantification with both β-actin and GAPDH markers, but there was still no difference in the amount of GIP expression.

In addition, to clarify whether GIP overexpression affects the proliferation of HT-22 cells, we performed the CCK-8 assay for 3 days at 24 h intervals under normal conditions. The role of GIP in protecting neuronal cells has not been proven. However, there was no difference in the GIP protein levels between mock and HT-22-overexpressing GIP cells. These results indicate that the proliferation of mock and GIP-overexpressing HT-22 cells does not differ (Supplementary Figure S1A). This may be caused by the rapid secretion and decomposition of GIP after protein synthesis. To verify this postulation, we treated transfected cells with a protein trafficking inhibitor, brefeldin A (BFA), for 3 h to block GIP secretion. Following this, we compared the total GIP protein levels. After blocking GIP secretion, the GIP protein band showed a significant difference between mock and GIP-transfected cell lines in Western blot analysis (Figure 2C). This result implies that the effects of GIP are maximised in the presence of a specific stimulus.

Then, we assessed whether GIP levels change during glutamate-induced neuronal cell death. We treated HT-22 cells with 5 mM glutamate and found that the GIP mRNA expression levels increased in GIP-overexpressing HT-22 cells (Figure 2D). Similarly, the GIP protein levels gradually increased in each group in a dose-dependent manner (Figure 2E). Consistent with Figure 2C, the difference in GIP protein levels was noticeable between mock and HT-22-overexpressing GIP cells after BFA treatment for 3 h (Figure 2E). This result implies that it is difficult to detect GIP as a secretory hormone under normal conditions. Hence, glutamate stimulation is a suitable method for evaluating the role of GIP. In addition, the increase in GIP expression following glutamate treatment was similar between mock and GIP-overexpressing cells. However, in GIP-overexpressing cells, GIP expression increased remarkably upon treatment with 5 mM glutamate. Following this, we assessed whether the overexpression of GIP under excessive glutamate conditions causes HT-22 cell state changes. The viability of GIP-overexpressing HT-22 cells almost doubled in the viability test (Figure 2F). This result suggests that GIP can protect against glutamate-mediated excitotoxicity by increasing its own expression.

To determine the effect of GIP on programmed cell death triggered by excessive glutamate, we checked the percentage of dead cells with annexin V–PI staining by the gating line we randomly assigned, as shown in Figure 2G (Figure 1C and Supplementary Figure S2A,B). The results indicated that GIP overexpression enhances cell survival through the attenuation of programmed cell death triggered by excessive glutamate. Morphologically, shrunken cell bodies and disappeared neurites were observed in glutamate-stimulated HT-22 cells upon microscopic analysis. The number of these cell types was much higher in the GIP-overexpressing HT-22 cells than in the mock cells (Figure 2H). Moreover, the immunofluorescence intensity of GIP expression noticeably differed between the GIP-overexpressing and mock cells (Figure 2I). Thus, GIP improves cell viability under excessive glutamate conditions.

### 3.3. Glutamate-Induced Neuronal Cell Death Is Distinct from Apoptosis

We aimed to clarify the type of cell death induced by excessive glutamate and reveal the distinct mechanism by which GIP promotes cell survival in mouse hippocampal HT-22 cells. To identify the type of programmed cell death associated with glutamate-induced HT-22 cell death, first, we examined apoptosis-related signalling pathways in HT-22 cells after glutamate treatment.

**Figure 3:**
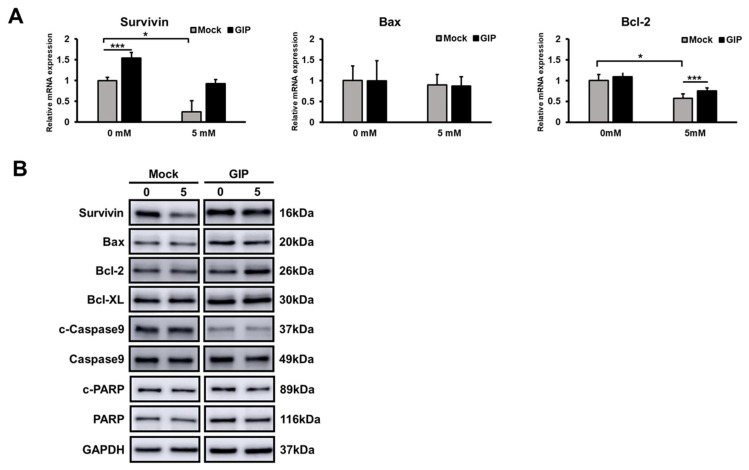
Glutamate-induced neuronal cell death is distinct from apoptosis. (**A**) Relative mRNA levels of apoptosis-related markers in HT-22 cells were evaluated by qRT-PCR in the presence or absence of 5 mM glutamate for 6 h. The graph shows the expression level of each apoptosis-related marker relative to that of GAPDH. Data are presented as the means ± SDs (*n* = 6). * *p* < 0.1 and *** *p* < 0.001. (**B**) Western blot analysis of apoptotic markers in mock or GIP-overexpressing, vector-transfected HT-22 cells treated with 5 mM glutamate for 6 h.

Interestingly, the expression and activation patterns of apoptotic markers were not consistent with the well-defined features of apoptosis under normal conditions. Accordingly, we investigated whether glutamate treatment could alter the levels of apoptotic markers in the control group. However, increased neuronal damage following glutamate treatment did not also cause apoptotic marker activation in the mock group. The mRNA levels of Bax, an apoptosis activator, did not differ significantly between the mock and GIP-overexpressing HT-22 groups (Figure 3A). Meanwhile, after treatment with 5 mM glutamate, the mRNA levels of antiapoptotic markers (Bcl-2), which were decreased in the 5 mM glutamate-treated group, were definitely higher in the GIP-overexpressing HT-22 cells than in the mock group (Figure 3A). The Bcl-2 protein level in mock HT-22 cells did not change even after glutamate treatment; however, it increased in GIP-overexpressing HT-22 cells. It may be a worthwhile consideration to maintain homeostasis and the recovery system.

Hence, we additionally checked the protein expression levels of cleaved forms of caspase-3 and -9, which represent the apoptosis pathway. In the case of caspase-3, the expression level of total caspase-3 was slightly lower in the normal GIP-overexpressing HT-22 cells, but there was no remarkable difference in the mock cells upon glutamate treatment (Supplementary Figure S4A). Additionally, the protein expression of cleaved caspase-3 was not detected and the inactivated caspase-3 (total caspase-3) band was observed as one band. In order to confirm whether this phenomenon is due to glutamate-induced cell death, which does not activate caspase-3, 2 μM of staurosporine (STS), a well-known apoptosis inducer, was treated as a positive control. As can be seen from the BF images, staurosporine-treated HT-22 cells showed a completely different morphology from cell death in which apoptosis is in progress (Supplementary Figure S4C). Unlike apoptosis, there were no branches and very rounded shapes in glutamate-induced HT-22 cells. Moreover, cleaved caspase-3 activated when apoptosis occurred was not observed in glutamate-induced cell death. (Supplementary Figure S4B). Once again, this result could be related to the fact that glutamate-induced HT-22 cell death is a completely different mechanism from apoptosis, and HT-22 cells lack activated caspase-3 in glutamate-induced cell death [37,38,39]. The caspase-9 expression level also did not show a significant increase, even upon glutamate treatment, as with caspase-3 (Figure 3B). Given the lower caspase-9 expression level in GIP-overexpressing HT-22 cells, we inferred that GIP also has an antiapoptotic effect under normal conditions. However, we further assessed the function of GIP and the type of cell death induced by glutamate in neurons. Finally, we assessed poly (ADP-ribose) polymerase 1 (PARP-1) cleavage to clarify whether ferroptosis is a type of parthanatos, a caspase-independent regulated cell death pathway. Although PARP-1 cleavage is a well-known specific apoptotic marker [40], some studies have recently reported that PARP-1 could induce another form of cell death, namely, parthanatos (i.e., PARP1-dependent cell death). Several studies have revealed that glutamate-induced excitotoxicity and those kinds of ischaemic diseases could be triggered by parthanatos [41,42]. Hence, we assessed the PARP-1 protein level; however, contrary to expectations, no significant differences were noted between either cell groups under normal conditions (Figure 3B). These data indicate that glutamate-induced cell death is not deeply associated with apoptosis or parthanatos.

### 3.4. GIP Reduces Glutamate-Induced Neuronal Oxidative Stress in HT-22 Cells

**Figure 4:**
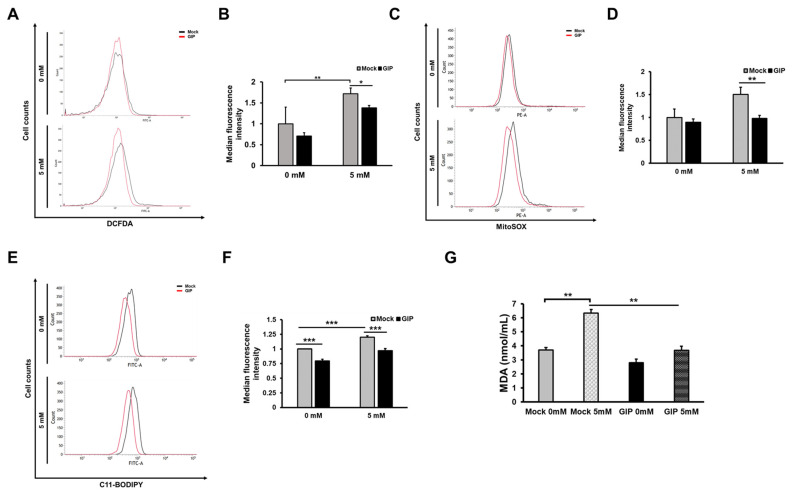
GIP protects neuronal HT-22 cells from glutamate-induced cell death by reducing oxidative stress and lipid peroxidation. All transfected HT-22 cells were exposed to 5 mM glutamate for 6 h. The levels of intracellular ROS (**A**), mitochondrial ROS (**C**) and lipid peroxidation (**E**) were measured by flow cytometric analysis after staining with DCFDA, MitoSOX and C11-BODIPY, respectively. The median fluorescence intensity of each flow cytometric result was statistically analysed: (**B**) intracellular ROS, (**D**) mitochondrial ROS and (**F**) lipid peroxidation. (**G**) Levels of MDA in HT-22 cells treated with 5 mM glutamate for 6 h were measured using TBARS assay. Untreated normal HT-22 cells were used as the negative control. Data are presented as the means ± SDs (*n* = 6). * *p* < 0.05, ** *p* < 0.01 and *** *p* < 0.001.

Glutamate-induced oxidative stress is associated with the dysregulation of mitochondrial function and a series of processes that consequently cause cell death in many neurodegenerative diseases [43]. To further examine how GIP improves cell viability in the presence of glutamate-induced damage regardless of apoptosis, we assessed the effects of GIP on cellular ROS levels, which cause oxidative stress and mitochondrial dysfunction [44]. Excessive glutamate results in ROS accumulation and lipid peroxidation, which could be critical factors for glutamate-induced neuronal damage [19,24]. Initially, we measured intracellular ROS levels by flow cytometry with CM-H2DCFDA staining. Compared with the control, the levels of intracellular ROS induced by treatment with 5 mM glutamate were attenuated in GIP-overexpressing HT-22 cells (Figure 4A,B). This indicates that GIP effectively inhibits glutamate-induced intracellular ROS production. In addition, we analysed mitochondrial ROS production and lipid peroxidation, which are known as ferroptosis phenomena [45]. As expected, the overexpression of GIP reduced the glutamate-mediated production of mitochondrial ROS and lipid peroxidation. MitoSOX and C11-BODIPY were used to detect mitochondrial ROS production (Figure 4C,D) and lipid peroxidation (Figure 4E,F), respectively. Since we observed the lipid peroxidation was changed in glutamate, we examined whether glutamate influences malondialdehyde (MDA), a lipid peroxidation marker, also measured (Figure 4G). As a result, MDA level increased by about two times when glutamate was treated in the mock group, whereas in the GIP-overexpressing group, MDA expression was lower than in the mock from the basic state without glutamate and showed a slight increase even when glutamate was treated. GIP-overexpressing HT-22 cells showed reduced lipid peroxidation in the absence of glutamate; the distinction became remarkable in the presence of excessive glutamate. These results indicate that excessive glutamate induces detrimental oxidative stress, e.g., ferroptosis in neurons and that GIP helps decrease ferroptosis by suppressing ROS production and subsequent lipid peroxidation.

### 3.5. GIP Makes the Cell Cycle Run Stably under Glutamate-Induced Stress Conditions

**Figure 5:**
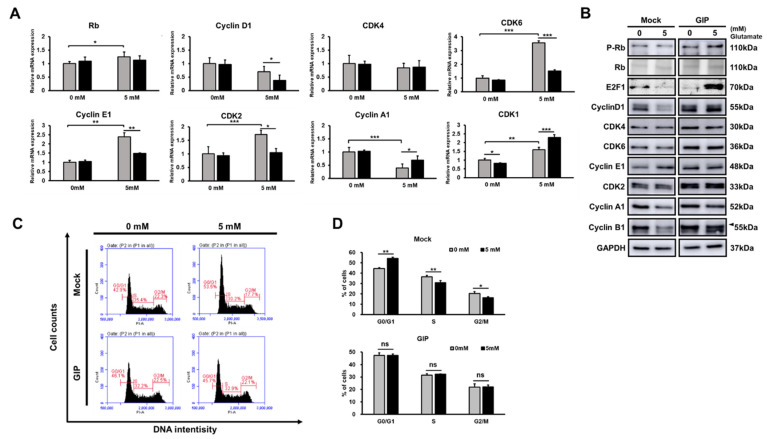
GIP prevents glutamate-induced cell cycle arrest. Transfected HT-22 cells were stimulated with 5 mM glutamate for 24 h. (**A**) The results of qRT-PCR analysis of relative mRNA levels of cell cycle markers. (**B**) Western blot analysis of protein expression levels of cell cycle-related factors. (**C**) Cells were stained with PI, and flow cytometric analysis was performed to evaluate the cell cycle. (**D**) The bar graph displays the percentage of each cell phase summarised from three independent experiments following PI staining (bottom panel). Data are presented as the means ± SDs (*n* = 3). * *p* < 0.05, ** *p* < 0.01 and *** *p* < 0.001.

To clarify the cell state under glutamate-induced neuronal cell damage conditions, we assessed the effect of glutamate treatment on the progression of the cell cycle through propidium iodide (PI) staining and measured the mRNA and protein levels of cell cycle-related factors. In particular, the mRNA expression levels of cyclin E1, cdk6 and cdk2 increased and that of cyclin A1 decreased after treatment with 5 mM glutamate in the control (Figure 5A). In addition, the protein levels of cyclin D1, A1 and B1 decreased, while cyclin E1 was accumulated in the mock group after treatment with 5 mM glutamate (Figure 5B). Most GIP-overexpressing HT-22 cells did not show any remarkable cell cycle arrest at specific points, even after glutamate treatment. Compared with the control, most GIP-overexpressing HT-22 cells showed higher basal protein levels of the indicated cell cycle-related factors. Furthermore, those two groups had a different tendency in terms of E2F transcription factor 1 (E2F1) and phospho-Rb (p-Rb) (Figure 5B). p-Rb combines with E2F factors and blocks their ability to facilitate the expression of genes that encode products responsible for S-phase progression [46]. Under normal conditions, GIP-overexpressing HT-22 cells showed lower E2F1 expression levels than the control; however, the opposite result was observed after treatment with 5 mM glutamate. Hence, we scrutinised the cell cycle phase by flow cytometric analysis with PI staining. An increase in the proportion of the G1 phase and a decrease in the S and G2/M phases were noted in 5 mM glutamate-treated vehicle HT-22 cells. These results imply that the stress induced by excessive glutamate causes G1/S arrest in HT-22 cells. In contrast, the cell cycle of GIP-overexpressing HT-22 cells was not affected by glutamate treatment (Figure 5C, 5D). The cell cycle arrest in glutamate-induced HT-22 cell death was not affected by GIP overexpression. The results suggest that GIP alleviates neuronal damage caused by excessive glutamate, which is involved in G1/S cell cycle arrest. As shown in Figure 4 and Figure 5, ROS accumulation could have resulted in G1/S arrest or vice versa [47]. The results proved that GIP inhibits G1/S arrest by lowering ROS levels under glutamate-induced neuronal toxicity conditions.

### 3.6. GIP Ameliorates Glutamate-Induced Ferroptosis through the Suppression of MAPK Activation

**Figure 6:**
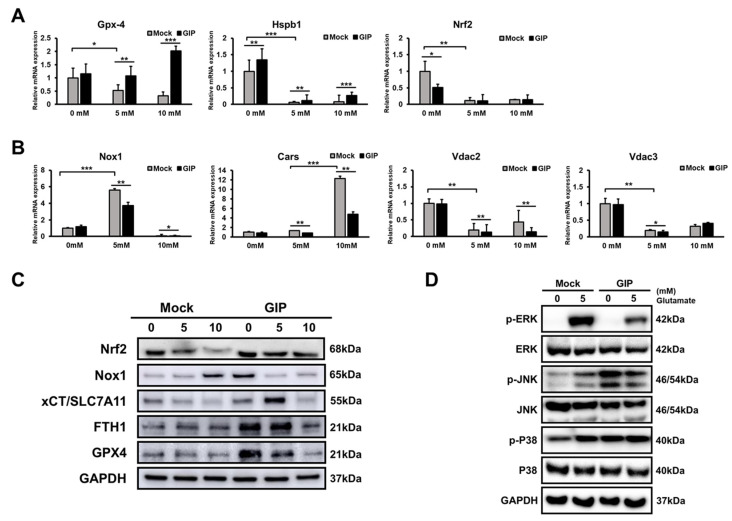
GIP reduces glutamate-induced ferroptosis and MAPK activation. (**A**,**B**) Transfectants were treated with the indicated concentrations of glutamate for 6 h, and then, mRNA levels of positive (**A**) and negative markers (**B**) of ferroptosis were assessed by qRT-PCR. (**C**) Western blot analysis was preformed to detect anti-ferroptosis factors in mock and GIP-overexpressing HT-22 cells. All samples were treated with the indicated glutamate concentrations (5 and 10 mM) for 6 h and then analysed. (**D**) The cells were treated with 5 mM or without glutamate for 9 h and the phosphorylation and total protein levels of MAPKs in each group were assessed by Western blotting. Data are presented as the means ± SDs (*n* = 3). * *p* < 0.05, ** *p* < 0.01 and *** *p* < 0.001.

As glutamate-induced cell death shows similar features to ferroptotic cell death [48], we compared the levels of ferroptosis-associated proteins between mock and GIP-overexpressing, vector-transfected HT-22 cells after glutamate treatment for 6 h. Notably, we used a much higher concentration of glutamate than that used in previous studies to clearly detect glutamate-triggered ferroptosis pathways. Upon treatment with 5 and 10 mM glutamate for 6 h, the relative mRNA levels of negative regulators of ferroptosis (glutathione peroxidase 4 (Gpx4), nuclear factor erythroid-2-related factor 2 (Nrf2) and heat shock protein family B (small) Member 1 (Hspb1)) remarkably increased in GIP-overexpressing HT-22 cells (Figure 6A). On the other hand, the levels of ferroptosis activators (NADPH oxidase 1 (Nox1), cysteinyl-tRNA synthetase (Cars) and voltage-dependent anion channel (Vdac2/3) significantly decreased when GIP was overexpressed (Figure 6B). Interestingly, the protein levels of Nrf2, xCT/SLC7A11, and Gpx4, which are considered to inhibit ferroptosis [49], were extremely expressed even upon glutamate treatment in GIP-overexpressing HT-22 cells but decreased or showed no change in control cells. Ferritin heavy chain 1 (FTH1), which also inhibits ferroptosis, was significantly increased in the GIP-overexpressing group [50]. On the other hand, mock and GIP-overexpressing (O/E) groups showed the opposite tendencies at the Nox1 level as a ferroptosis inducer [51]. In the GIP O/E group, which definitely plays a protective role in ferroptosis, the basic expression level of Nox1 was high at 0 mM, but the expression level of nox1 decreased when neuronal damage was induced by glutamate, and the GIP effect was clearly observed (Figure 6C). As shown in Figure 4 and Figure 6A–C, glutamate-induced neuronal cell death is considered to be ferroptosis and not apoptosis. In addition, GIP suppresses ferroptosis, which occurs as a result of excessive glutamate. Many studies have revealed that under glutamate-induced neuronal toxicity conditions, HT-22 cells undergo cell death associated with cellular ROS accumulation [52,53]. Notably, MAPK pathways are crucial for the regulation of cell survival, cell proliferation and programmed cell death; their activation is closely associated with cellular ROS levels, in addition to glutamate-induced cell death. [54,55]. Moreover, GIP plays many roles in the regulation of the metabolism via the MAPK pathway [56]. GIP can prevent β-amyloid-induced neuronal cell death by inhibiting the ERK signalling pathway in HT-22 cells, indicating the association between GIP and MAPK signalling pathways during cellular apoptosis [57]. However, to the best of our knowledge, no previous study has demonstrated an association between GIP and MAPKs for neuronal survival under glutamate-induced oxidative stress. As expected, MAPK phosphorylation was suppressed in GIP-overexpressing HT-22 cells under glutamate-induced oxidative stress (Figure 6D). In particular, the phosphorylation of p38 was gradually upregulated by glutamate in a dose-dependent manner in the control group but was not detected clearly in the GIP-overexpressing group. The ERK protein was weakly expressed under normal conditions in both the groups. Unlike GIP-overexpressing HT-22 cells, mock cells showed increased ERK phosphorylation after treatment with 5 mM glutamate. ERK phosphorylation levels remained unchanged in GIP-overexpressing HT-22 cells treated with 5 mM glutamate. On the other hand, the expression level of JNK was high in GIP-overexpressing HT-22 cells under normal conditions but decreased until it became the same as that in the control group at high concentrations of glutamate. These results indicate that GIP alleviates glutamate-induced ferroptosis through the suppression of MAPK activation.

## 4. Discussion

GIP has been primarily considered an incretin hormone that has been receiving attention for its roles in metabolism, such as its effects on insulin secretion, fat accumulation and bone formation [58,59,60]. Recently, the neuroprotective effect of GIP against brain damage or neurodegenerative diseases has been reported in some studies conducted with GIP analogues [28,57,61]. However, there are some differences between native GIP and its analogues in terms of stability and activity. Endogenous GIP is rapidly degraded by dipeptidyl peptidase-4 (DPP-IV). Some studies have proved the effects of GIP through treatment with long-lasting GIP analogues or agonists that are resistant to DPP-IV [62,63]. Most researchers have prepared and used modified GIP to study the effects of GIP on neuronal diseases; however, the exact functions and mechanisms of natural GIP have not yet been elucidated. Furthermore, these modified GIP (1–30) analogues have lesser activity than full-length GIP (1–42), showing lower potency and weaker binding to GIPR [64,65]. In the present study, we investigated the biological functions of endogenous GIP in neuronal cells by the overexpression of genes instead of treatment with GIP analogues. Interestingly, to the best of our knowledge, this is the first in vitro study to use endogenous GIP-overexpressing neuronal cells. Several studies have demonstrated that GIP-overexpressing mice show increased bone mass [66] and reduced obesity [67]. Other studies have used GIP receptor knockout mice to assess neuronal function [68,69]. As GIP is expressed in the hippocampus and promotes progenitor cell proliferation [29], we thought that the neuron-specific overexpression of GIP could reveal the neuroprotective role of native full-length GIP by overcoming the decrease in GIP expression levels due to DPP-IV degradation. Although we established GIP-overexpressing HT-22 cells, the increased degree of GIP protein (Figure 2B) was much lower compared to the mRNA difference (Figure 2A). This also highlights the possibility that the protein expression level of GIP would decrease during the process of rapid release. This could be checked by treating BFA as a protein trafficking inhibitor in the established HT-22 cell line. Another interesting factor is that BFA and glutamate, which are known to induce ER stress [70,71], showed similar results (Figure 2B). It suggests that GIP is definitely involved in glutamate-induced oxidative stress. Maybe GIP exerts a greater protective effect when there is a specific toxic injury, such as excessive glutamate, than under normal conditions.

Oxidative stress is considered a major cause of cell death and dysfunction in numerous diseases. According to the pathogenesis of many neurodegenerative diseases, brain regions, particularly the cortical and hippocampal regions, are susceptible to oxidative stress; chronic stress could accelerate cognitive loss/synaptic plasticity impairment and neurodegeneration [72]. Glutamate was first identified as a neurotransmitter in the brain. It mainly exists in the presynaptic terminal and is transported to the synaptic cleft by synaptic vesicles [73]. However, it has been reported that excessive glutamate production in the brain is toxic to neurons; this is known as excitotoxicity. Exogeneous glutamate induces oxidative stress due to an imbalance of the GSH/oxidised glutathione (GSSG) ratio and subsequent mitochondrial dysfunctions [19]. Glutamate excitotoxicity may advance during deleterious events, e.g., as a secondary injury after a traumatic injury [74] or during ischaemia [75]. Generally, the protective roles of GIP have been defined under specific damage, such as ischaemia or chronic inflammation response [76]. For creating similar circumstances in vitro, we established an excessive glutamate environment in mouse hippocampal HT-22 cells.

In Figure 1D,E, there was a significant increase in GIP expression levels upon glutamate stimulation in mouse hippocampal cells. This phenomenon suggests that GIP is associated with glutamate-induced cell death in HT-22 cells in some way. Considering previous findings, we hypothesised that GIP overexpression plays a specific role in the protective mechanism against excessive glutamate. Thus, we decided that the HT-22 cell line would be effective when overexpressing GIP as a target gene using a neuron-specific promoter. Notwithstanding, there was no change in cell proliferation in either the mock or GIP-overexpressing HT-22 group. These data indicate that GIP functions more efficiently during neuronal toxicity not when homeostasis is maintained (Figure 2B,C). In addition to glutamate treatment also increasing GIP expression in mock vector-transfected cells as control, GIP-overexpressing cells showed higher viability at the same glutamate concentration (Figure 2F,G). However, the type of glutamate-induced cell death remains ambiguous. First, we noted glutamate-induced oxidative stress in HT-22 cells without any regard to representative apoptotic factors, such as caspase-3, caspase-9 and Bcl-2. We found that fatal toxicity caused by glutamate induces G1/S arrest in the HT-22 cell cycle; however, GIP makes the cell cycle run stably even in the presence of glutamate-induced neuronal damage (Figure 5).

Next, we assessed the exact indicators underlying glutamate-induced cell death. GIP-overexpressing cells could downregulate the lipid peroxidation level (Figure 4E–G). The stress resulting from ROS production and lipid peroxidation explains that our findings related to glutamate-induced oxidative stress are associated with ferroptosis. Especially, as lipid peroxidation is the important factor in ferroptosis [77]. Hence, it would be more meaningful to assess our previous findings (Figure 2 and Figure 4). General cell death can be assessed by CCK-8 assay and microscopy images to check whether the cells have a special morphology, such as axons of nerve cells (in this case, HT-22 cells). In order to distinguish it from apoptosis in this process, we also used an apoptosis inducer called staurosporine (STS) and showed a clear difference in morphology. In the process of ferroptosis, the association with PI staining has been revealed, but annexin V staining has not yet been accurately assessed. However, the present study was conducted using both only PI staining and annexin V-PI double staining (Figure 2G and Supplementary Figure S2A) as the FACS experimental technique to observe the degree of basic cell death caused by mitochondrial membrane damage and morphological changes.

Before identifying the specific cell death mechanism, the protective ability of GIP was assessed after excessive glutamate treatment (Figure 2F–I). As mentioned, many studies have demonstrated glutamate-induced apoptosis in HT-22 cells [55,78]. Hence, we initially assessed the role of GIP in the apoptosis of neuronal cells; however, some of the results did not meet our expectations (Figure 3). To understand how GIP protects cells against excitotoxicity, we used samples with numerous cell death-related markers. In addition, we measured several ROS levels to determine the cause of the distinctive cell viability tendency (Figure 4A). It is likely that GIP can inhibit glutamate-induced oxidative stress. An important point is the protective effect of GIP upon a very high concentration of glutamate (e.g., 10 mM) could induce a higher intracellular calcium concentration with higher calcium-dependent protease activation and reduced protein levels.

Focusing on the neuronal cell death pathway triggered by excitotoxicity, we additionally checked the PARP activation part of apoptosis to reveal glutamate-induced toxicity. However, the features of glutamate-induced neuronal cell death were quite different not only from apoptosis but also parthanatos (Figure 3B). Although ferroptosis is yet to be explored clearly, the growing tendencies explained that the type of glutamate-induced cell death in HT-22 cells was ferroptosis. As shown in Figure 6, ferroptosis resulting from extracellular glutamate-induced toxicity evokes MAPK phosphorylation, and its activation as a result of cell death could be prevented when GIP is overexpressed. As ferroptosis is a type of iron-dependent cell death that is characterised by GSH imbalance, mitochondrial impairment, DNA oxidation and lipid peroxidation [14], and includes the MAPK signalling pathway [51], we confirmed and explained those related markers (Figure 6A–D). In summary, we demonstrated that glutamate-induced neuronal death is characterised as ferroptosis and not apoptosis and that GIP defends HT-22 cells from glutamate-induced ferroptosis by increasing their antioxidant capability, which is mediated by Gpx-4 and Nrf2 activity.

It is known that the GIP receptor is a G-protein-coupled receptor (GPCR) which regulates cell cycle progression and inhibits Raf-1 activated ERK activity through RAP-1/B-Raf/ERK pathways [79]. The MAPK pathway involves JNK, p38 and Erk1/2 and has been reported to play multiple roles. The regulation of this pathway, including three subgroups of MAPKs (i.e., ERKs, JNKs and p38 MAPKs), is important for determining cell fate (both cell growth and cell death). The detrimental results of the continued activation of MAPK pathways include the excessive production of MAPK-regulated genes, rampant proliferation and unexpected cell death [80]. However, the activation of the MAPK pathway is essential for neuronal differentiation, synapse plasticity, cell survival and cell behaviour [81,82]. Hence, the suppression of MAPK activity would be a crucial strategy for preventing ferroptosis caused by glutamate-induced oxidative stress and for treating neurodegenerative diseases [83]. Interestingly, GIP suppresses p38 MAPK and JNK for inhibition of apoptosis [84], and facilitates the proliferation of some cells through the activation of MAPK pathways at the same time [84,85]. However, the present results indicated that GIP suppresses ferroptosis through the suppression of MAPK signalling pathways. In summary, GIP provides a neuroprotective environment against glutamate-induced excitotoxicity by enhancing cell survival, avoiding cell cycle arrest, and inhibiting ferroptotic cell death by regulating the MAPK pathway, ferroptotic mechanisms and ROS production. However, how GIP regulates both mitochondrial ROS and ferroptosis remains unclear. Further in vivo analysis is required to identify the main molecular mechanism by which GIP can affect glutamate-mediated neuronal stress.

## 5. Conclusions

**Figure 7:**
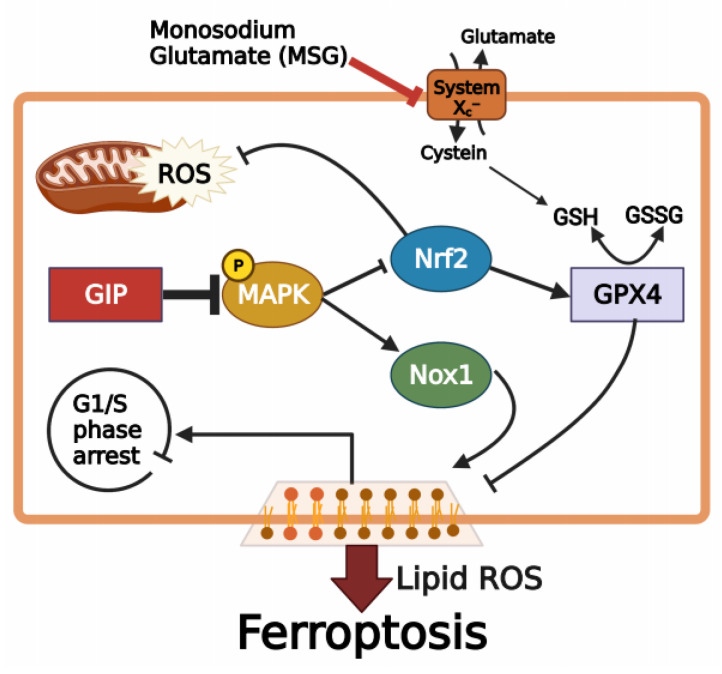
Schematic depicting the regulation of Nrf2 and Nox1 by GIP during glutamate-induced ferroptosis in HT-22 cells. Excessive glutamate inactivates GPX4 function to induce lipid ROS accumulation. Overexpression of GIP attenuates ferroptosis by regulating Nrf2 and Nox1 ferroptosis factor through MAPK signal pathway. G1/S phase arrest induced by ferroptosis can be normalised in GIP-overexpressing HT-22 cells.

In this study, we found that the type of programmed cell death caused by excessive glutamate-induced neurotoxicity in mouse hippocampal HT-22 cells is ferroptosis and not apoptosis or parthanatos. Moreover, the neuroprotective role of GIP was evident in the form of a normal cell cycle without any phase arrest and ROS-scavenging effects in the present study. We provide the evidence that GIP suppresses glutamate-induced ferroptosis through the MAPK signalling pathways in HT-22 cells (Figure 7). This suggests that GIP significantly protects neuronal cells from various diseases that are mostly related to oxidative stresses. Nevertheless, the detailed mechanisms by which GIP regulates the activation of upstream target kinases or proteins in MAPK signalling pathways to inhibit glutamate-induced ferroptosis remain unclear and require further study.

## Figures and Tables

**Table 1:** List of gene-specific primer sequences used for real-time qRT-PCR.

Name	Forward Sequence	Reverse Sequence	Al^a^(bp)
GIP (NM_008119.2)	CTCTTTGCCCAAGAGCCTCA	ATCAGAAGGTCCCTCAGCACA	92
CDK1 (NM_007659.4)	CAGAACTGGCCACCAAGAAG	TTGTTAGGAGTGCCCAGAGC	131
CDK2 (NM_183417.3)	ATGGACGGAGCTTGTTATCG	CATCCTGGAAGAAAGGGTGA	133
CDK4 (NM_009870.4)	GGCCCTCAAGAGTGTGAGAG	CCTCCTTAACAAGGCCACCT	131
CDK6 (NM_009873.3)	AGAAGTCCTGCTCCAGTCCA	AAGAGGCTTTCT GCGAAACA	131
Cyclin D1 (NM_001379248.1)	TTGACTGCCGAGAAGTTGTG	CCACTTGAGCTTGTTCACCA	136
Cyclin E1 (NM_007633.2)	CCCTCTGACCATTGTGTCCT	ACCTGCTGTGGGTACTGAGG	136
Cyclin A1 (NM_001305221.1)	TCCACTTCCTGCTGGATTTC	CTGAACCAAAATCCGTTGCT	133
Nox1 (NM_172203.2)	CTCCAGCCTATCTCATCCTGAG	AGTGGCAATCACTCCAGTAAGGC	166
VDAC2 (NM_011695.2)	TCGGCAAAGCTGCCAGAGACAT	GTCTCCAAGGTCCCGCTAACTT	195
VDAC3 (NM_001198998.1)	GCCTTTGAAGGTTGGCTTGCTG	GAGCCTCCAAACTCAGTGCCAT	190
CARS (NM_013742.5)	GGGCTCTGCTGGAGAACATT	AGGGCATGACTGTTGACTCG	178
GPX4 (NM_008162.4)	CGCTCCATGCACGAATTCTC	GTGACGATGCACACGAAACC	126
HSPB1 (NM_013560.2)	GCTCACAGTGAAGACCAAGGAAG	TGAAGCACCGAGAGATGTAGCC	137
Nrf2 (NM_010902.4)	CAGCATAGAGCAGGACATGGAG	GAACAGCGGTAGTATCAGCCAG	151
Bax (NM_007527.3)	GGCGAATTGGAGATGAACTG	CAAAGTAGAAGAGGGCAACCAC	201
Bcl-2 (NM_009741.5)	TCGCCCTGTGGATGACTGA	CACTTGTGGCCCAGGTATG	240
Caspase-3 (NM_001284409.1)	ATGGGAGCAAGTCAGTGGAC	CGTACCAGAGCGAGATGACA	177
Caspase-9 (NM_015733.5)	GGCGGAGCTCATGATGTCTGTG	TTCCGGTGTGCCATCTCCATCA	313
PARP (NM_007415.3)	CTCTCCCAGAACAAGGACGAAG	CCGCTTTCACTTCCTCCATCTTC	190
GAPDH (NM_001289726.1)	TGAGGCCGGTGCTGAGTATGTCG	CCACAGTCTTCTGGGTGGCAGTG	348

## Data Availability

The data presented in this study are available in the manuscript.

## Supplementary Materials

The following are available online at https://www.mdpi.com/article/10.3390/antiox11020189/s1, Figure S1: The proliferation of HT-22 cells after GIP-transfected in HT-22 cells; Figure S2: Evaluated cell death between the GIP-overexpressing and mock HT-22 cells with or without MSG treatment; Figure S3: Lipid peroxidation assessment in HT-22 cells in glutamate dose-dependent manner; Figure S4: Glutamate-induced HT-22 cell death is a type of cell death notably different from apoptosis; Figure S5: Quantification of the Western blot data in figure 2B, 2C, and 2E, respectively; Figure S6: Quantification of the Western blot data in Figure 3B; Figure S7: Quantification of the Western blot data in Figure 5B; Quantification of the Western blot data in figure 6C; Figure S9: Quantification of the Western blot data in figure 6D.
