# Supplementary material for: Protective Effect of GIP against Monosodium Glutamate-Induced Ferroptosis in Mouse Hippocampal HT-22 Cells through the MAPK Signaling Pathway
Source: Antioxidants (Basel). 2022 Jan 19;11(2):189. doi: 10.3390/antiox11020189 (PMC8868324; doi:10.3390/antiox11020189)
Supplement: Supplementary file 1 [file antioxidants-11-00189-s001.zip › antioxidants-1526067-supplementary.pdf]

Supplementary Materials

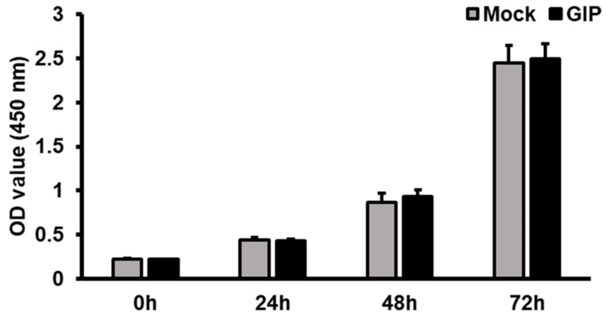

Figure S1. The proliferation of HT-22 cells after GIP-transfected in HT-22 cells. CCK-8 assay for cell proliferation for 0-72 h between mock and GIP-transfected HT-22 cells. Data are presented as mean ± SD (n = 3).

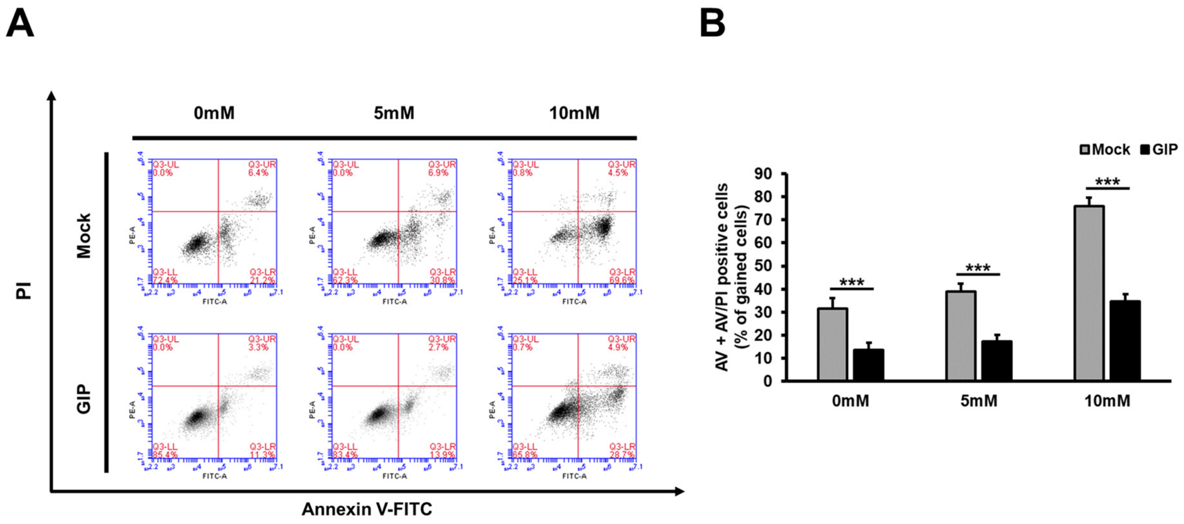

Figure S2. Evaluated cell death between the GIP-overexpressing and mock HT-22 cells with or without MSG treatment. (**A**) Transfected HT-22 cells were treated with indicated concentrations (0, 5, 10 mM) of MSG for 12 h. The programmed cell death was then evaluated using flow cytometric analysis with annexin V and PI staining. This result is representative of three independent experiments. (**B**) Flow cytometry data were quantified and statistically analyzed among groups. Data are presented as the means ± SDs (n = 3). *** $p < 0.001$, compared with the mock group of each glutamate treated cell.

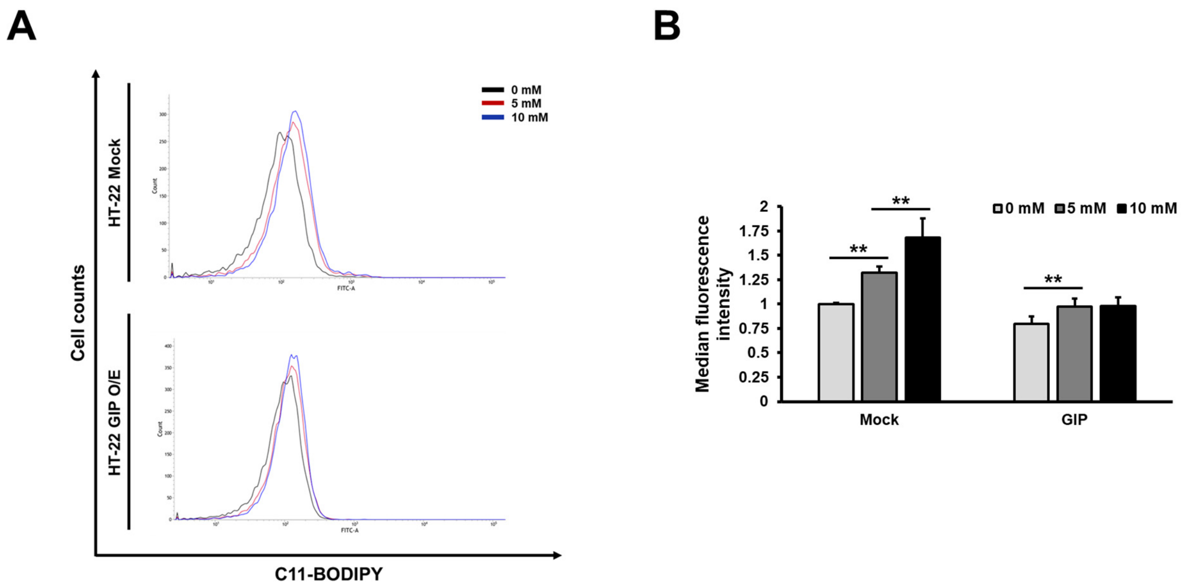

Figure S3. Lipid peroxidation assessment in HT-22 cells in glutamate dose-dependent manner. (**A**) FACS analysis of C11-BODIPY[581/591] as a lipid peroxidation indicator in the glutamate-treated vehicle and GIP-overexpressing (GIP O/E) HT-22 cells. The C11-Bodipy staining was performed at 6 h after glutamate treatment. Black, Red, and Blue indicate 5 mM, 10 mM of glutamate treatment conditions, respectively. (**B**) FITC-A fluorescence intensity was defined as lipid ROS positive cells, which is indicated in the right bar graph. Data are presented as the means ± SDs (n = 3). ** p < 0.01.

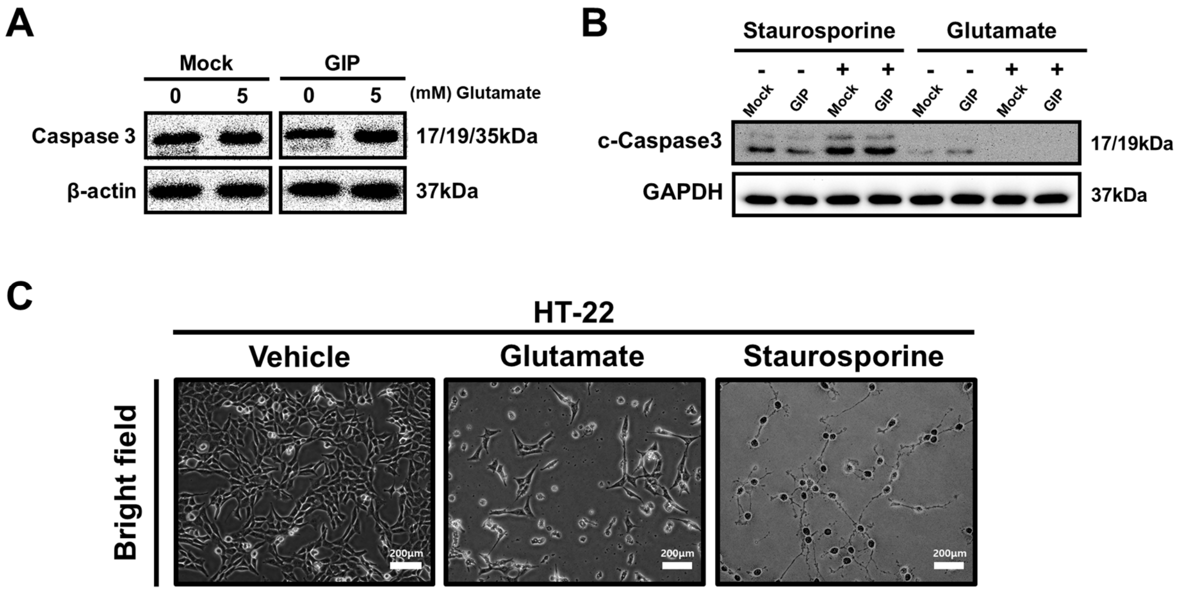

Figure S4. Glutamate-induced HT-22 cell death is a type of cell death notably different from apoptosis. (**A**) Caspase 3 was detected in mock and GIP-overexpressing HT-22 cells with or

without 5 mM glutamate treatment by western blot. (**B**) Western blot of the HT-22 cells with 1 μM STS or 5 mM glutamate treatment with cleaved-caspase 3 antibodies in HT-22 transfected cells. Cleaved caspase 3 was not detected in glutamate-induced HT-22 cells. (**C**) Bright-field images of the normal HT-22 cells, 5 mM glutamate-induced HT-22 cells for 24 h, and 1 μM staurosporine(STS) for 2h in normal HT-22 cells.

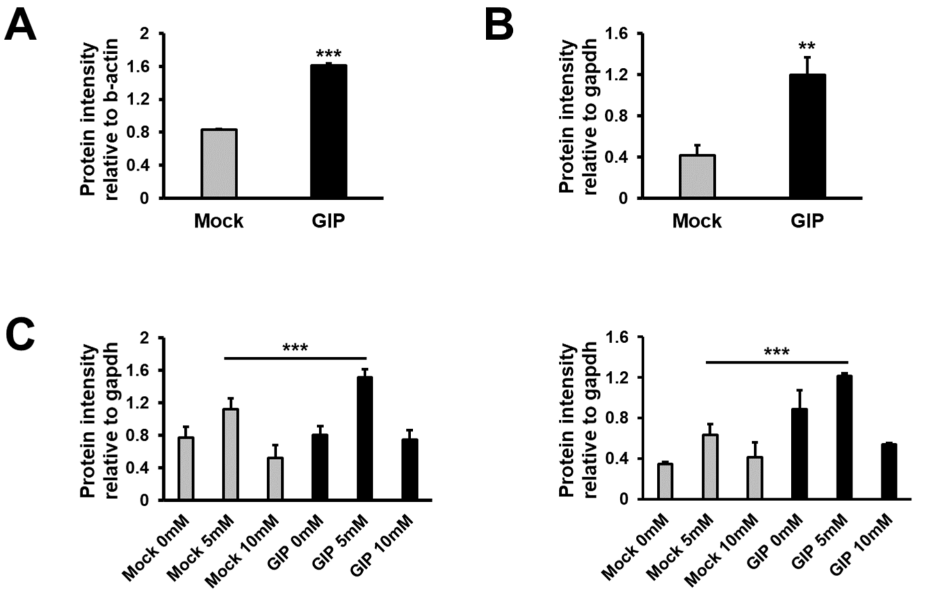

Figure S5. Quantification of the Western blot data in Figure 2B, 2C, and 2E, respectively. (**A**–**C**) GIP protein intensity was normalized with the housekeeping gene and presented as a ratio relative to the area density of the housekeeping gene. The area density of each band was measured with the ImageJ program. Data are presented as the means ± SDs (n = 3). ** $p < 0.01$ and *** $p < 0.001$.

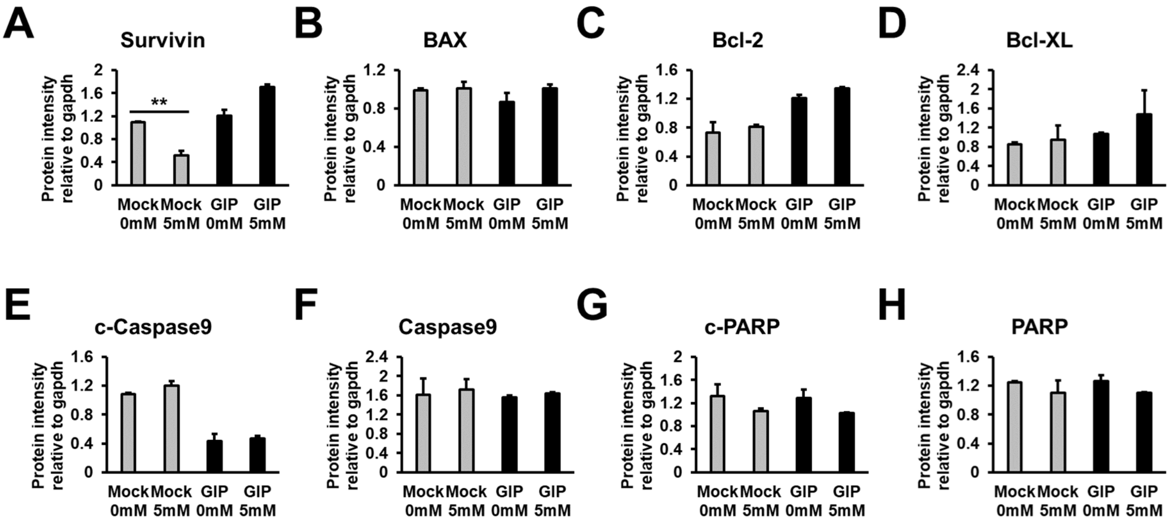

Figure S6. Quantification of the Western blot data in Figure 3B. (**A**–**H**) The protein density of apoptosis markers was calculated with the ImageJ program. All western blot data were normalized with the GAPDH and presented as a ratio relative to the area density of the GAPDH. Data are presented as the means ± SDs (n = 3). ** $p < 0.01$.

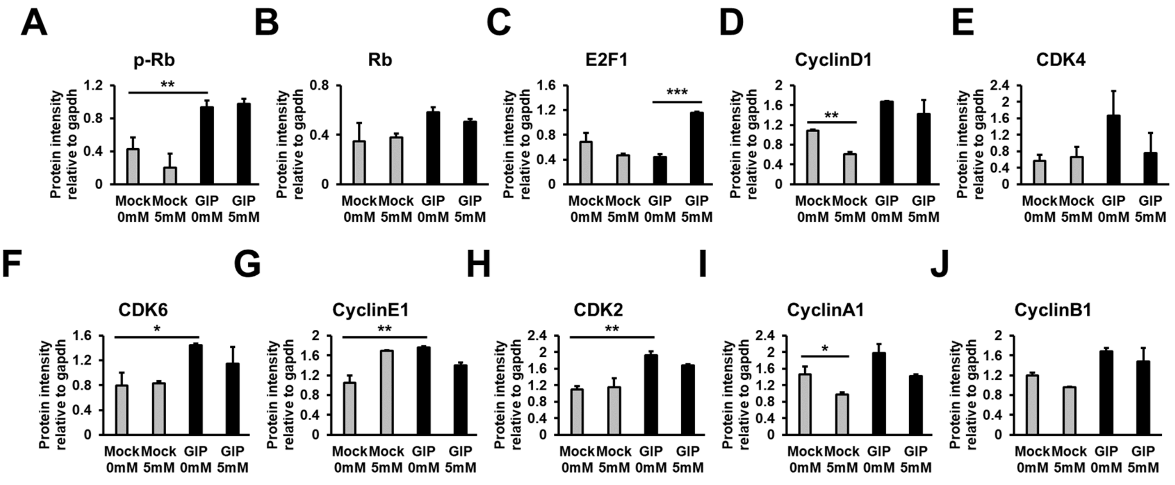

Figure S7. Quantification of the Western blot data in Figure 5B. (**A**–**J**) The area density of each band of the cell cycle markers was evaluated with the ImageJ program. The data were normalized with the GAPDH and presented as a ratio relative to the area density of the GAPDH. Data are presented as the means ± SDs (n = 3). * $p < 0.05$, ** $p < 0.01$ and *** $p < 0.001$.

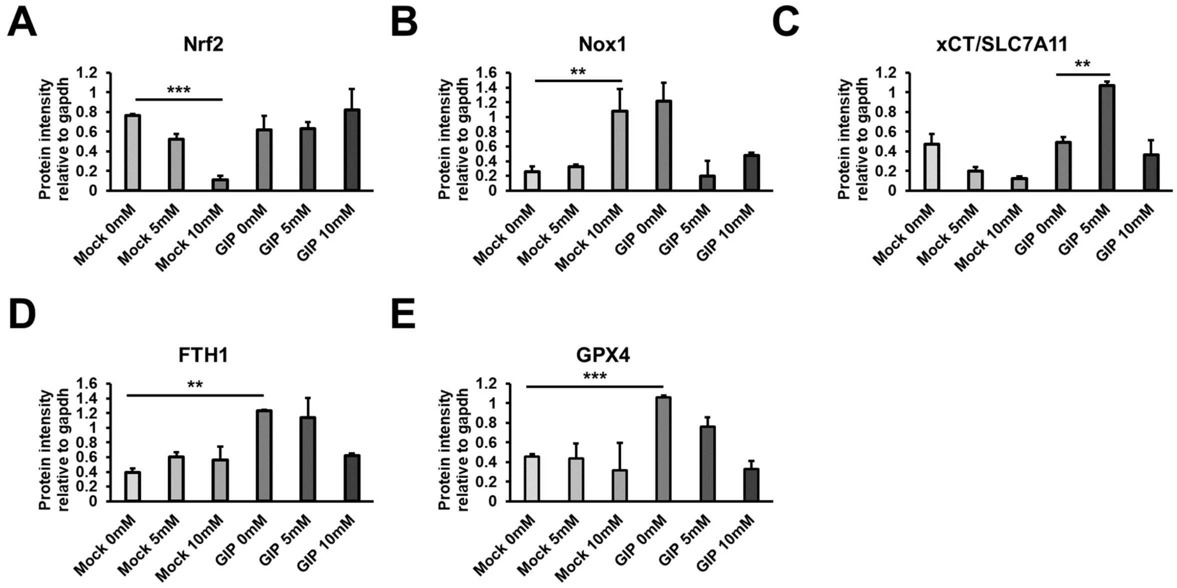

Figure S8. Quantification of the Western blot data in figure 6C. (**A**–**E**) The protein intensity of ferroptosis-related markers in each band was calculated with the ImageJ program. The data were normalized with the GAPDH and presented as a ratio relative to the area density of the GAPDH. Data are presented as the means ± SDs (n = 3). **p < 0.01 and ***p < 0.001.

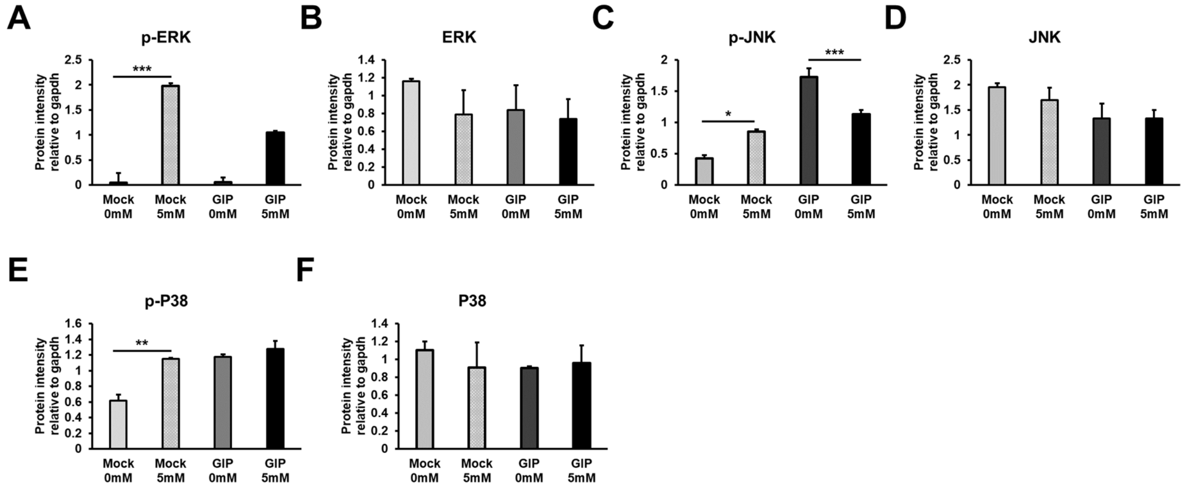

Figure S9. Quantification of the Western blot data in figure 6D. (**A**–**F**) The relative protein expression level of MAPK signaling markers was analyzed through ImageJ. The data were normalized with the GAPDH and presented as a ratio relative to the area density of the GAPDH. Data are presented as the means ± SDs (n = 3). *p < 0.05, **p < 0.01 and ***p < 0.001.
